# Chidamide Combined With Doxorubicin Induced p53-Driven Cell Cycle Arrest and Cell Apoptosis Reverse Multidrug Resistance of Breast Cancer

**DOI:** 10.3389/fonc.2021.614458

**Published:** 2021-03-02

**Authors:** Lixia Cao, Shaorong Zhao, Qianxi Yang, Zhendong Shi, Jingjing Liu, Teng Pan, Dongdong Zhou, Jin Zhang

**Affiliations:** Third Department of Breast Surgery, Tianjin Medical University Cancer Institute and Hospital, National Clinical Research Center for Cancer, Key Laboratory of Cancer Prevention and Therapy, Clinical Research Center for Cancer, Tianjin, China

**Keywords:** breast cancer, histone deacetylase, chidamide, doxorubicin, drug resistance

## Abstract

The multidrug-resistant (MDR) phenotype is usually accompanied by an abnormal expression of histone deacetylase (HDAC). Given that HDAC is vital in chromatin remodeling and epigenetics, inhibiting the role of HDAC has become an important approach for tumor treatment. However, the effect of HDAC inhibitors on MDR breast cancer has not been elucidated. This study aim to demonstrate the potential of chidamide (CHI) combined with the chemotherapy drug doxorubicin (DOX) to overcome chemotherapeutic resistance of breast cancer *in vitro* and *in vivo*, laying the experimental foundation for the next clinical application. The results showed that, CHI combined with DOX showed significant cytotoxicity to MDR breast cancer cells *in vitro* and *in vivo* compared with the CHI monotherapy. The cell cycle distribution results showed that CHI caused G0/G1 cell cycle arrest and inhibited cell growth regardless of the addition of DOX. At the same time, annexin V staining and TUNEL staining results showed that CHI enhanced the number of cell apoptosis in drug-resistant cells. The western blot analysis found that p53 was activated in the CHI-treated group and combined treatment group, and then the activated p53 up-regulated p21, apoptosis regulator recombinant protein (Puma), and pro-apoptotic protein Bax, down-regulated the apoptotic proteins Bcl-xL and Bcl-2, and activated the caspase cascade to induce apoptosis.

## Introduction

Doxorubicin (DOX) is an anthracycline widely used as the first-line treatment of breast cancer (1, 2). The pharmacological effect of this drug is to intervene between gene base pairs of DNA, interfere with gene transcription, and inhibit the synthesis of DNA and RNA in tumor cells. With time, the cancer cells become resistant to drugs. Once drug resistance develops, the effect of the drugs decreased significantly (3). The drug resistance of breast cancer cells is the main reason for the failure of chemotherapy and the recurrence of the disease, and it is one of the problems that need to be solved urgently in clinical practice. Drug-resistant cells respond to chemotherapy drugs through different mechanisms, such as strong DNA damage repair ability, cell cycle change, apoptosis retardation, epigenetic modifications, and abnormal activation of multiple signaling pathways, including PI3K/AKT, Notch, Hedgehog, p53/p21, and Wnt pathways (4–6). Among them, the p53/p21 pathways are important factors for tumor cell resistance to chemotherapy drugs (7–9).

In recent years, epigenetic abnormalities have become an important indicator of tumor development and progression. Histone is one of the basic components of chromosomes in the human body (10). Its acetylation is important in the development of tumors. When HDAC is overexpressed in cells, it causes acetylation imbalance inducing tumorigenesis (11). Given that HDAC plays a vital role in chromatin remodeling and epigenetics, inhibiting the role of HDAC has become an important approach to tumor therapy. In fact, HDAC1, HDAC2, HDAC3, HDAC6, and HDAC7 have been shown to be overexpressed in breast cancer (12–15). Studies found that the down-regulation of HDAC inhibited the proliferation and survival of tumor cells in drug-resistant breast cancer cells and delayed the progression of breast cancer (16).

Chidamide (CHI) is the first subtype-selective histone deacetylase inhibitor (HDACi) independently developed and synthesized in China, which can selectively inhibit HDAC1, HDAC2, HDAC3 in class I and HDAC10 in class IIb (17). It is used more in breast cancer because of its good curative effects, few adverse reactions, strong targeting, and easy administration. In combination therapy, multiple oncogenic signaling pathways can also be targeted simultaneously, thereby increasing the possibility of overcoming drug resistance in difficult-to-treat advanced breast cancer (18, 19).

In this study, the efficacy of CHI was analyzed in MDR breast cancer cell lines. In addition, CHI had a synergistic sensitization effect with DOX. The combined therapy downregulated the expression of HDAC1, activated p53 and released p21, inhibited cell proliferation, and induced MDR cell cycle arrest and apoptosis. This study demonstrated the potential of CHI combined with the chemotherapy drug DOX to overcome chemotherapeutic resistance of breast cancer, laying the experimental foundation for the next clinical application.

## Materials and Methods

### Cell Culture

Human breast cancer cell line Cal51 and its MDR counterpart CALDOX were both obtained from Dr. Ernesto Yague (Imperial College London, UK) (20). Human breast cancer cell line MCF-7 and its MDR counterpart MCF-7/A02 were both obtained from Professor Dongsheng Xiong (Institute of Hematology, PUMC, Tianjin, China) (20). All the cells were maintained in the RPMI-1640 medium (Corning Incorporated), supplemented with 10% fetal bovine serum (Corning Incorporated) and 1% penicillin-streptomycin (Corning Incorporated) at 37°C in an atmosphere with 95% air and 5% CO_2_. CHI was derived from Chipscreen Biosciences (Shenzhen, China) and dissolved in DMSO at a final concentration of 1 mM. DOX was purchased from Rhawn (Shanghai, China) and dissolved in DMSO at a final concentration of 1 mM.

### Cell Viability Analysis

The cell counting kit-8 (CCK-8, Dojindo, Japan) was used to evaluate the effects of DOX or CHI alone or in combination on cell viability. The cells were seeded in a 96-well plate with a density of 2×10^4^ to 4×10^4^ cells/ml and 100 μl complete medium per well. After 3 days of treatment with different concentrations of CHI or DOX or a combination of the two, 10 μl of CCK-8 reagent was added to each well and incubated for 2 h. The absorbance detection was measured at 450 nm using a microplate reader (Rayto, USA). Based on the results, the concentration of the drug that inhibited cell growth by 50% (IC50) was calculated. For drug combination experiments (21), CalcuSyn software (Biosoft, Cambridge, UK) was used to calculate the combination (CI) values based on median dose effect analysis after the combinations of a range of DOX and CHI concentrations. The CI values between 0.1 and 0.9 indicated different degrees of synergism: CI values between 0.9 and 1.1 indicated additive, whereas CI values >1.1 are indicated antagonistic effects.

### Crystal Violet Staining

The cells were seeded in six-well plates (2 × 10^5^ cells/well) and treated with DOX (2 μM for CALDOX and 0.4μM for MCF-7/A02) and CHI (6μM for CALDOX and 4μM for MCF-7/A02) for 1 week at 37°C. The resistant clones were fixed with 4% paraformaldehyde (Servicebio, Wuhan, China) and stained with 0.4% (w/v) crystal violet (Solarbio, Beijing, China) and counted. The crystal violet remaining in the cells was dissolved in 33% (v/v) acetic acid (Solarbio, Beijing, China) and quantified by measuring the optical density at 592 nm (22).

### EDU Staining

Cells at logarithmic growth stage were inoculated in 24-well plates with 1× 10^4^-2 ×10^4^ cells per well and cultured to normal growth stage. The EDU program used Cell-Lighetm EDU Apollo488 In Vitro Kit (RiboBio, Guangzhou, China) and was observed by Axioplan 2 microscope.

### Cell Cycle Analysis

After 48 h of treatment, the cells were fixed in 70% ethanol overnight at 4°C, washed twice with PBS, treated with RNase A for 30 min at 4°C and stained with propidium iodide (Sigma–Aldrich, Merck KGaA, final concentration, 20 µg/ml) for 30 min at 4°C. The samples were analyzed using a BD FACSCanto II (Becton Dickinson, San Jose, CA, USA) flow cytometer to determine the proportion of cells at each stage of the cell cycle using flow cytometry software (ModFit LT, Verity Software House, Inc., Topsham, ME, USA).

### RNA Isolation and Real-Time Quantitative PCR

The total cellular RNA was isolated using an RNA extraction solution (Wuhan Goodbio Technology Co., Ltd.) following the manufacturer’s protocol. A RevertAid First-Strand cDNA Synthesis Kit (Thermo) was used to generate cDNA with 2 µg RNA. The real-time quantitative PCR (RT-qPCR) was performed using SYBR Green I (Takara, Dalian, China) and detected using an ABI SDS7900 Real-time PCR System (Applied Biosystems). Specific gene primers were synthesized by GenePharma (Shanghai, China) (**Table 1**). The RT-qPCR conditions were as follows: one cycle at 94°C for 30 s and 45 cycles at 94°C for 5 s and 60°C for 30 s. The melting curve analysis was from 60 to 95°C at a 0.3°C increase per 15 s. The results were analyzed using the 2^−ΔΔCt^ method; and ΔΔCt = Cttarget gene of sample − Ctβ-actin of sample − (Cttarget gene of control − Ctβ-actin of control). All experiments were repeated three times.

**Table 1:** Primers for quantification measurements of mRNA expression.

Gene	Forward	Reverse
Bcl-2	CTGGGAGAACAGGGTACGATAA	GGCTGGGAGGAGAAGATGC
Bax	TCATCCAGGATCGAGCAGG	TGTCCACGGCGGCAAT
Caspase-3	AGGCAGGCGACGAGTT	TTCCCATAGAGTTCCACAAA

### Annexin V Staining

Cell apoptosis was detected using Annexin V−FITC/PI Assay Kit (ImmunoWay, Texas, USA), according to the procedure recommended by the manufacturer. The cells (1 × 10^5^) were washed twice with PBS and suspended in 100 μl binding buffer followed by staining with 5 μl Annexin V−FITC for 30 min in a dark room. 5 μl PI was added for 5 min, and the total volume was finally replenished to 250−300 μl with binding buffer. The fluorescence was detected using a flow cytometer (BD FACSCanto II). The quantitative values showed the average percentage of annexin V−positive cells (lower right quadrant, both in early apoptosis; upper right quadrant, late apoptosis), of three independent experiments

### Western Blot Analysis

After 48h of treatment, the cells were lysed using RIPA buffer (Solarbio, Beijing, China). The supernatant was collected by centrifugation at 12,000 rpm for 10 min at 4°C, and the total protein in the specimen was quantified using BCA kit (Solarbio, Beijing, China). Proteins in equal amounts were separated by sodium dodecyl sulfate-polyacrylamide gel electrophoresis and electrotransferred onto PVDF membranes (Millipore, USA), and then the membranes were blocked with 5% blotting-grade milk. The membranes were incubated overnight at 4°C with primary antibodies rabbit anti-GAPDH (CST, 2118, 1:1,000 dilution), rabbit anti-HDAC1 (CST, 34589, 1:1,000 dilution), rabbit anti-histone H3 (acetyl K9, CST, 9649, 1:1,000 dilution), rabbit anti-histone H3 (acetyl K18, CST, 13,998, 1:1,000 dilution), rabbit anti-histone H3 (CST, 12230, 1:1,000 dilution), rabbit anti-p21 (ImmunoWay, YM3453, 1:1,000 dilution), mouse anti-p53 (ImmunoWay, YM3052, 1:2,000 dilution), rabbit anti-Puma (CST, 12450, 1:1,000 dilution), rabbit anti-Bcl-xL (CST, 2764, 1:1,000 dilution), rabbit anti-Bcl-2 (CST, 3498, 1:1,000 dilution), rabbit anti-caspase-7 (CST, 12827, 1:1,000 dilution), rabbit anti-cleaved-caspase-7 (CST, 8438, 1:1,000 dilution), rabbit anti-caspase-3 (CST, 9665, 1:1,000 dilution), rabbit anti-cleaved-caspase-3 (CST, 9664, 1:1,000 dilution), mouse anti-caspase-9 (CST, 9508, 1:1,000 dilution), rabbit anti-cleaved-caspase-9 (CST, 7237, 1:1,000 dilution) at 4°C overnight. The membranes were washed with Tris-buffered saline plus Tween 20 (TBST) for 30 min and incubated with secondary antibodies of horseradish peroxidase (HRP)-conjugated goat anti-mouse and anti-rabbit IgG (Servicebio, GB23301 and GB23303, respectively, 1:3,000 dilution) at room temperature for 1 h. Western blot signal detection was performed using SuperSignal West Pico chemiluminescent substrate (Pierce), following the manufacturer’s recommended instructions

### *In Vivo* Xenografts

The cells (1 × 10^7^) were suspended in 100 μl PBS containing 10% Matrigel (BD Biosciences) and injected into the mammary fat pad of 5-week-old female nude mice (SiPeiFu Company, Beijing, China). Tumor sizes were measured with a caliper every 3 days in two dimensions, and the tumor volume was calculated using the following formula: tumor volume (mm^3^) = 0.5 ×*ab* ^2^(*a* and *b* being the longest and shortest diameters of the tumor, respectively). Fourteen days after the cell injection, the tumor-bearing mice were randomly divided into four groups (five mice/group): 1) control group (normal saline), 2) DOX group (2 mg DOX per kg BW), 3) CHI group (5 mg CHI per kg BW), and 4) CHI+DOX group (5 mg CHI and 2 mg DOX per kg BW). The drugs were injected every 3 days and tumor volumes were monitored until the mice were euthanized. Subsequently, the tumors were collected to extract proteins and RNA. All mice were raised in accordance with the National Institutes of Health guidelines for laboratory animal care and use. The use of the animals in this study was approved by the Animal Care and Use Committee of Tianjin Cancer Hospital.

### TUNEL Assay

For the TUNEL assay *in vitro and in vivo*, cells were first fixed with 4% paraformaldehyde and then permeabilized with 1% Triton X-100. The TUNEL procedure was performed using the *in situ* cell death detection kit (Roche, Shanghai, China) and the cells were mounted in SlowFade Antifade with DAPI (Solarbio, Beijing, China) and viewed using a Zeiss Axioplan 2 microscope.

### Statistical Analysis

All data in this research were expressed as the mean ± standard deviation (SD) of three independent experiments. Line charts or corresponding bar graph were drawn by GraphPad Prism 7 software. Student’s *t* test was used when comparing the means of two groups. The one-way analysis of variance was used when comparing the means among more than two groups. *P* value less than 0.05 was considered statistically significant.

## Results

### Effects of CHI and DOX on the Viability of MDR Breast Cancer Cells

**Figure 1:**
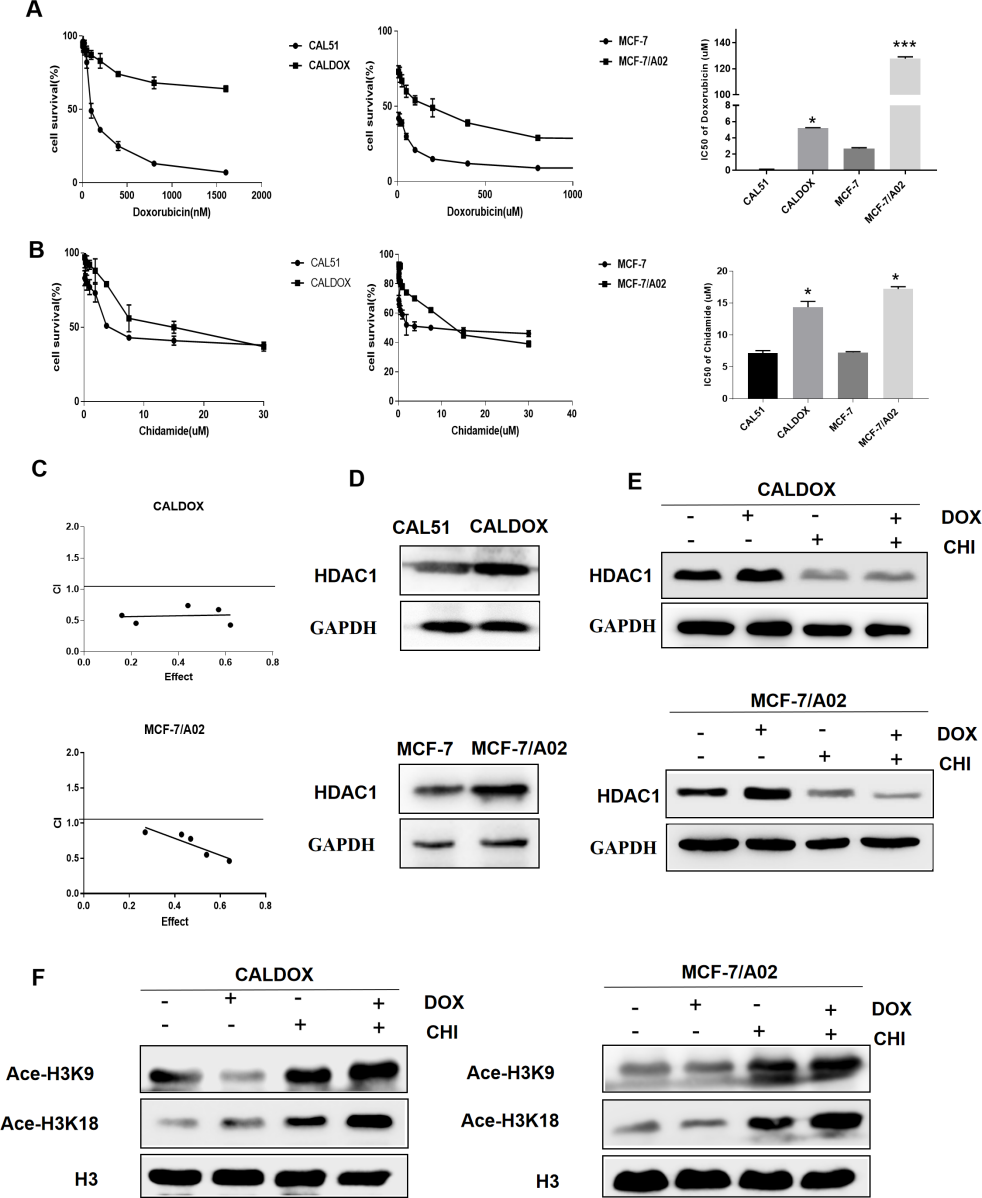
Effects of chidamide (CHI) and/or doxorubicin (DOX) on the viability and histone H3 acetylation of MDR breast cancer cells. **(A)** IC50 values of DOX of two pairs of human breast cancer cell lines and their multidrug-resistant (MDR) sublines. **(B)** IC50 values of CHI of two pairs of human breast cancer cell lines and their MDR sublines. **(C)** Cytotoxicity of CHI and DOX to CALDOX and MCF-7/A02 cells. **(D)** Expression of HDAC1 in sensitive and resistant cell lines. **(E)** Effects of CHI and DOX on HDAC1 expression in drug-resistant cells. **(F)** Effects of CHI and DOX on acetylation of H3K9 and H3K18 in drug-resistant cells. H3 was used as a loading control. The numerical values are expressed as mean ± standard deviation (SD) of three independent replicates. *P < 0.05, ***P < 0.001.

The MDR breast cancer cells CALDOX and MCF-7/A02 were derived from chemosensitive cell lines Cal51 and MCF-7, respectively. The chemosensitive and chemoresistant breast cancer cell growth was inhibited by CHI in a dose-dependent manner. The IC50 test results showed that the resistance of the two drug-resistant cell lines to DOX was 41.98 times and 47.58 times, respectively compared with their parental chemosensitive counterparts (**Figure 1A**). However, the resistance of the two drug-resistant cell lines to CHI was 1.8-fold and 1.9-fold, respectively (**Figure 1B**).The results showed that the chemoresistant cell lines showed no resistance to CHI. Next, the effect of the combination of CHI and DOX on cell viability was evaluated using median dose effect analysis. The CALDOX and MCF-7/A02 cells were treated with increasing concentrations of CHI, either alone or in combination with DOX at fixed ratios (DOX/CHI, 1:3 for CALDOX and 1:10 for MCF-7/A02) (**Table 2**). Living cells were detected using the CCK-8 proliferation method, and data were analyzed by GraphPad Prism software. CompuSyn software was used to evaluate the combined effect. The combination index (CI) value was 0.1–0.9 (**Figure 1C**), indicating that CHI and DOX had a synergistic effect in CALDOX and MCF-7/A02 cells.

**Table 2:** Cytotoxicity of chidamide (CHI) and doxorubicin (DOX) to MDR breast cancer cells.

**CALDOX**					
Doxorubicin (µM)	0.625	1.25	2.5	5	10
Chidamide (µM)	1.875	3.75	7.5	15	30
Cl	0.38	0.69	0.86	0.449	0.781
**MCF-7/A02**					
Doxorubicin (µM)	0.2	0.4	0.8	1.6	3.2
Chidamide (µM)	2	4	8	16	32
Cl	0.463	0.551	0.778	0.839	0.870

### Expression of HDAC1 in Breast Cancer and the Effect of CHI on Histone H3 Acetylation

The basic expression level of CHI target HDAC1 in MDR breast cancer cell lines and sensitive cell lines was investigated (23). As shown in **Figure 1D**, HDAC1 was expressed in sensitive cell lines and drug-resistant cell lines (CAL51, CALDOX, MCF-7, and MCF-7/A02), and the expression level of HDAC1 in drug-resistant cell lines was slightly higher than sensitive cell lines. Next, the acetylation of histone H3 lysine residue was measured to determine the inhibitory effect of CHI on HDAC. As shown in **Figure 1E**, CHI downregulated the expression of HDAC1 in drug-resistant cells, and significantly increased the acetylation of H3K9 and H3K18, regardless of the addition of DOX (**Figure 1F**).

### CHI Combined with DOX Inhibited the Proliferation and Induced Cell Cycle Arrest in MDR Breast Cancer Cells

**Figure 2:**
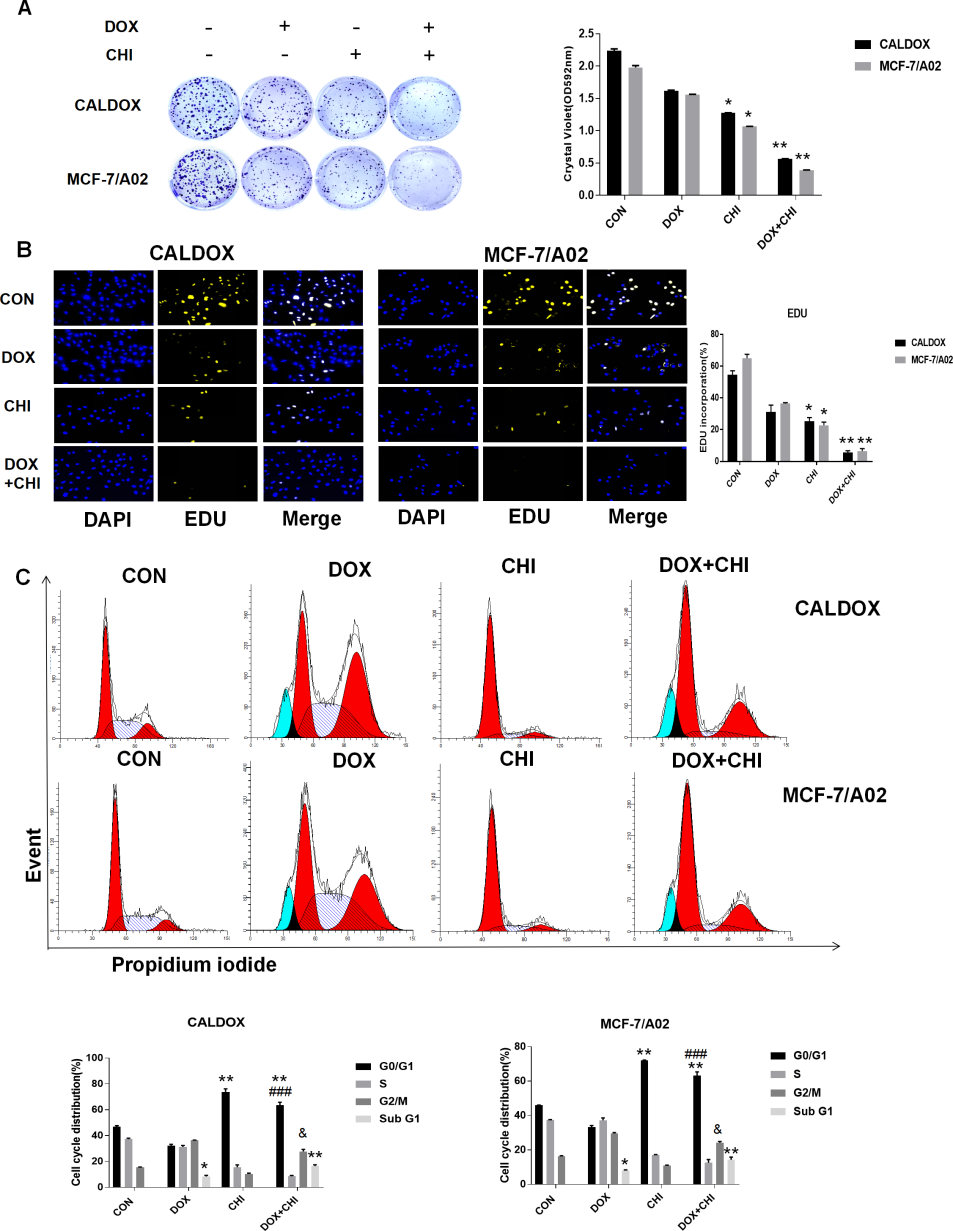
Effects of chidamide (CHI) and/or doxorubicin (DOX) on the proliferation and cell cycle of multidrug-resistant (MDR) breast cancer cells. **(A)** Drug resistance clonogenic assay confirmed the effect of CHI and/or DOX on cell proliferation. **(B)** EDU staining confirmed the effect of CHI and/or DOX on cell proliferation. **(C)** Effects of CHI and/or DOX on cell cycle. Numerical values are expressed as mean ± SD of three independent replicates. “*” indicates a significant difference compared with the control group (*P < 0.05, **P < 0.01),”#” indicates a significant difference compared with the DOX-treated group (^###^P<0.001), and “&” indicates a significant difference compared with the CHI-treated group (^&^P<0.05).

To further evaluate the killing effect of CHI combined with DOX on chemotherapy-resistant breast cancer cells, cells were treated with DOX (2 μM for CALDOX and 0.4μM for MCF-7/A02) and CHI (6μM for CALDOX and 4μM for MCF-7/A02) for 7 days, As expected, based on crystal violet staining, the inhibitory effect was significantly higher in the combined group than in the monotherapy group (**Figure 2A**). The effect of CHI in combined with DOX on proliferation was confirmed. Furthermore, based on EDU staining, the number of EDU positive cells (yellow) and DAPI positive cells (blue) was visually measured. As shown in **Figure 2B**, the percentage of EDU incorporation in the combined medication group decreased significantly compared with the monotherapy group. The inhibitory effect of CHI on cell cycle was detected by flow cytometry. CALDOX and MCF-7/A02 cells were treated with CHI (0–10 mol/L) with increasing doses for 48h. CALDOX and MCF-7/A02 cells cycle arrest induced by CHI during G0/G1 phase (**Supplement Figure 1**). The cell cycle distribution of CALDOX and MCF-7/A02 cells exposed to DOX and CHI alone or in combination for 48h was analyzed by using flow cytometry. The proportion of G0/G1 phase cells prominently increased in the CHI-treated and combined medication group compared with the control group. In addition, the combined use of DOX and CHI significantly increased the percentage of cells at G2/M phase compared with the CHI-treated group, and the percentage of Sub G1 increased in the DOX-treated group and combination medication group (**Figure 2C**).

### Cell Apoptosis Induced by CHI Combined with DOX in MDR Breast Cancer Cells

**Figure 3:**
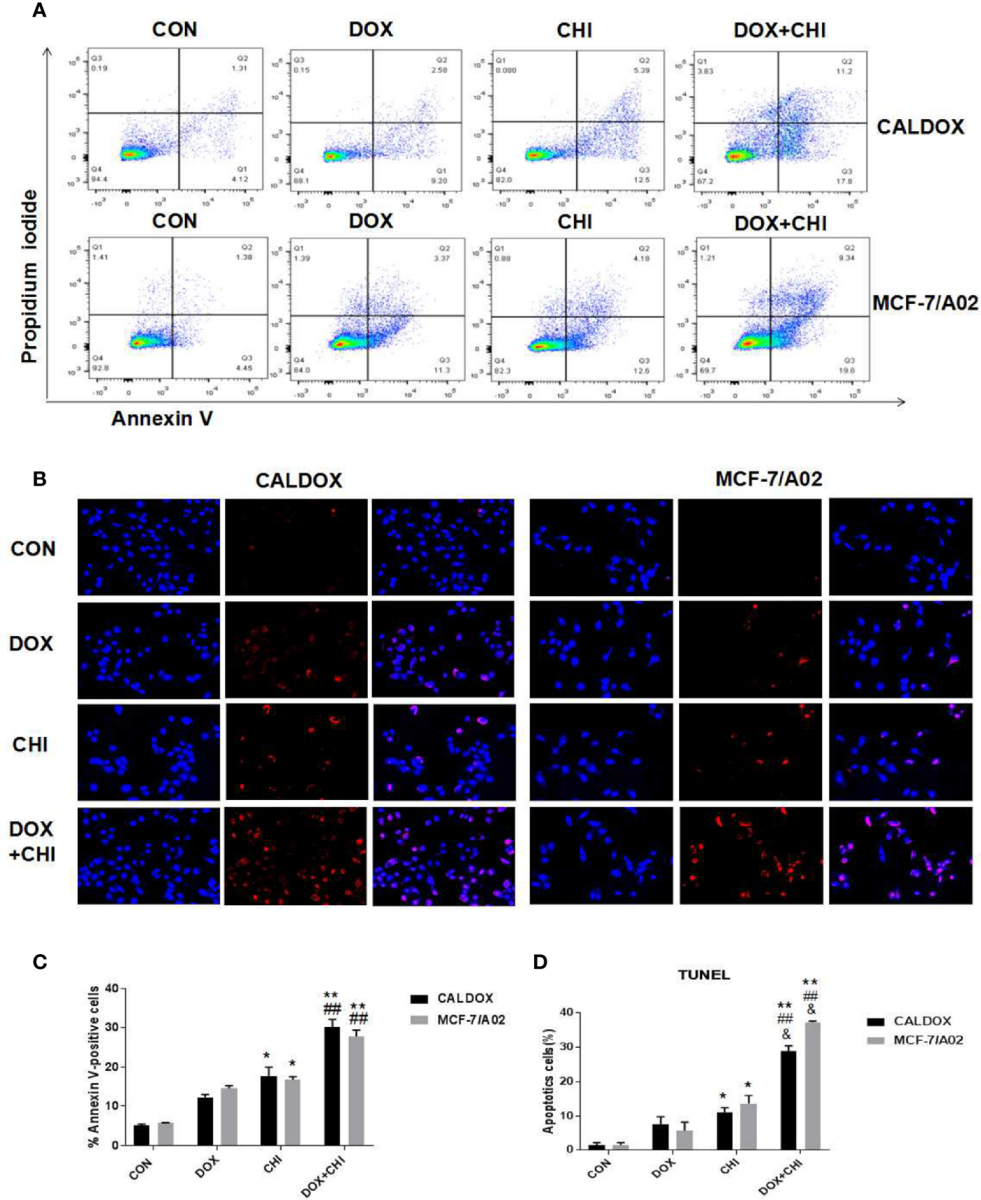
Effects of chidamide (CHI) and/or doxorubicin (DOX) on apoptosis of multidrug-resistant (MDR) breast cancer cells. **(A)** After treatment with CHI and/or DOX (48h), flow cytometry was used to detect apoptosis. Annexin V/PI staining was measured with flow cytometry. Representative plots of three independent experiments are shown. Quantitative values showed the average percentage of Annexin V-positive cells (lower right quadrant, both in early apoptosis; upper right quadrant, late apoptosis) of three independent experiments. **(B)** Apoptosis was determined using TUNEL staining assay. The number of TUNEL-positive cells (red) and DAPI-positive cells (blue) was visually measured. All samples were subjected to at least two biological replicate analyses, and three images of each replicate were obtained using a 20× objective to count TUNEL-positive cells and DAPI-positive cells. The percentage of TUNEL-positive cells was calculated as (TUNEL-positive cells/total cells) × 100. The numerical values are expressed as mean ± S **(D)** of three independent replicates. “*” indicates a significant difference compared with the control group (*P < 0.05, **P < 0.01),”#” indicates a significant difference compared with the DOX-treated group (^##^P < 0.01), and “&” indicates a significant difference compared with the CHI-treated group (^&^P < 0.05).

Cell apoptosis was detected by flow cytometry to further explore the mechanism of cell death induced by CHI and DOX. The results showed that the apoptotic rate was significantly higher in the combined medication group compared with the control group and the DOX-treated group. In addition, the apoptotic rate was higher in the CHI-treated group compared with the control group (**Figure 3A**). Furthermore, the number of TUNEL-positive cells (red) and DAPI-positive cells (blue) was visually measured. As shown in **Figure 3B**, the percentage of TUNEL-positive cells increased significantly in the combined medication group. These results showed that CHI enhanced the apoptosis of DOX on CALDOX and MCF-7/A02 cells.

### CHI Combined with DOX Induced Cytotoxicity by Driving p53/p21 to Induce Cell Cycle Arrest and Promote Caspase-Dependent Apoptosis

**Figure 4:**
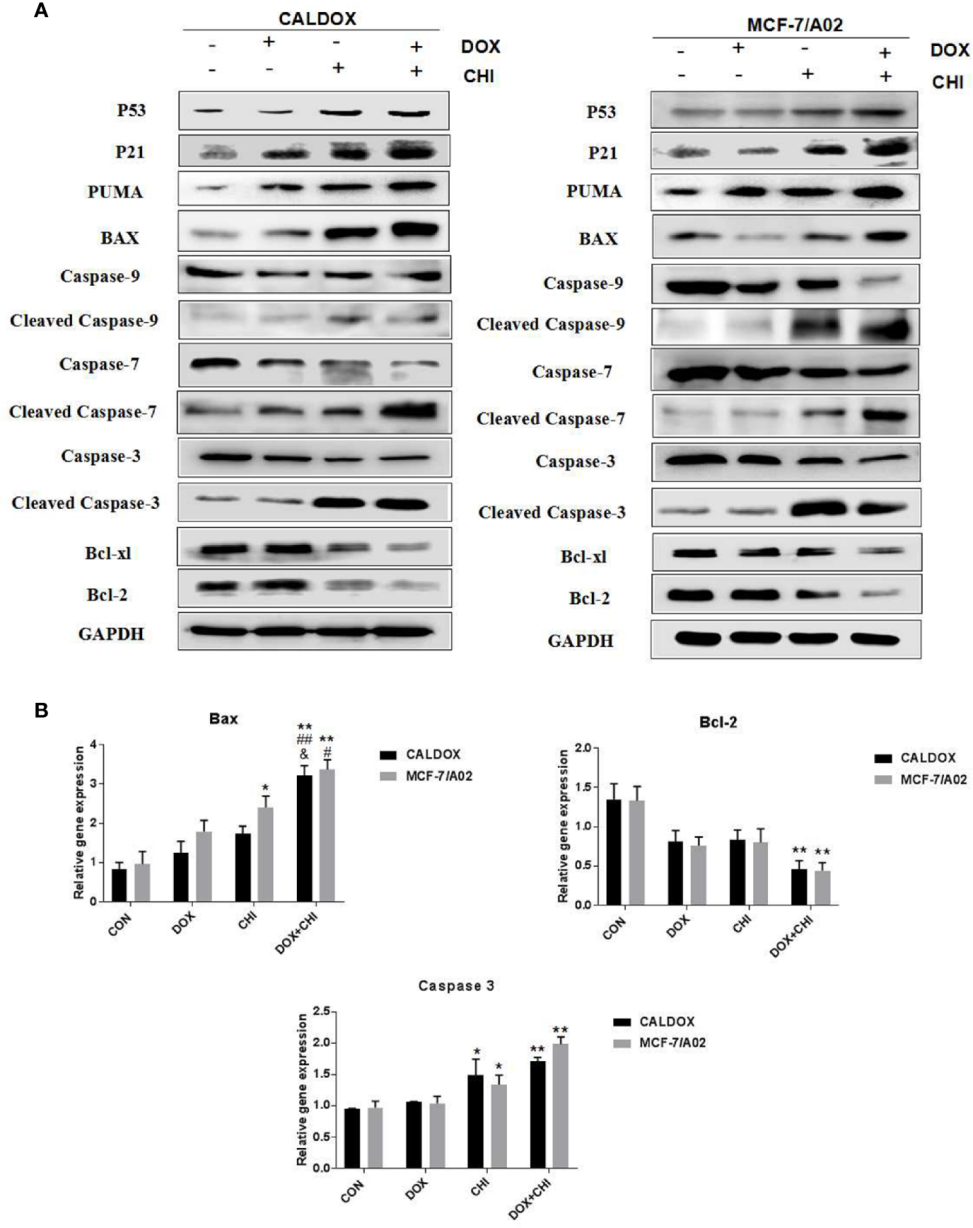
Chidamide (CHI) combined with doxorubicin (DOX) induced cytotoxicity by driving p53/p21 to induce cell cycle arrest and caspase-dependent apoptosis. Cells were treated with DOX and/or CHI for 48 h. **(A)** The western blot analysis showed that CHI combined with DOX treatment down-regulated Bcl-xl, Bcl-2, caspase-9, caspase-7, caspase-3 and up-regulated p53, p21, Puma, Bax, cleaved caspase-9, cleaved caspase-7, cleaved caspase-3 in CALDOX and MCF-7/A02 cells. GAPDH was used as a loading control. **(B)** Fold changes in Bcl-2, Bax and caspase-3 mRNA levels were detected using RT-qPCR in MDR cells. The numerical values are expressed as mean ± SD of three independent replicates. “*” indicates a significant difference compared with the control group (*P < 0.05, **P < 0.01), “#” indicates a significant difference compared with the DOX-treated group (^#^P < 0.05, ^##^P < 0.01), and “&” indicates a significant difference compared with the CHI-treated group (^&^P < 0.05).

The p53/p21 signaling pathway was often dysregulated in human cancers and associated with the resistance to standard anticancer therapies. Therefore, whether the cytotoxic effect of CHI combined with DOX on MDR breast cancer cells was due to the activation of the p53/p21 signaling pathway was further explored. The expression levels of p53, p21, caspase-3/7/9, and the Bcl family were further detected. After 48h of combined treatment with CHI and DOX, the western blot analysis showed that p53 and p21 were upregulated in the CHI-treated group and combined treatment group compared with the control group (**Figure 4A**), which might explain the mechanism of G0/G1 cell cycle arrest (24). The western blotting analysis showed that the levels of Bcl-xL, Bcl-2, caspase-9, caspase-7, and caspase-3 were downregulated and those of Puma, Bax, cleaved caspase-9, cleaved caspase-7, and cleaved caspase-3 were upregulated in the combined medication group compared with the control group (**Figure 4A**). According to RT-qPCR, when CALDOX and MCF-7/A02 cells were exposed to CHI and DOX, the relative gene expression of Bax, caspase 3 increased significantly and the relative gene expression of Bcl-2 decreased (**Figure 4B**)

### Effect of CHI Combined with DOX on Xenograft Tumor Growth of CALDOX Cells in Nude Mice

**Figure 5:**
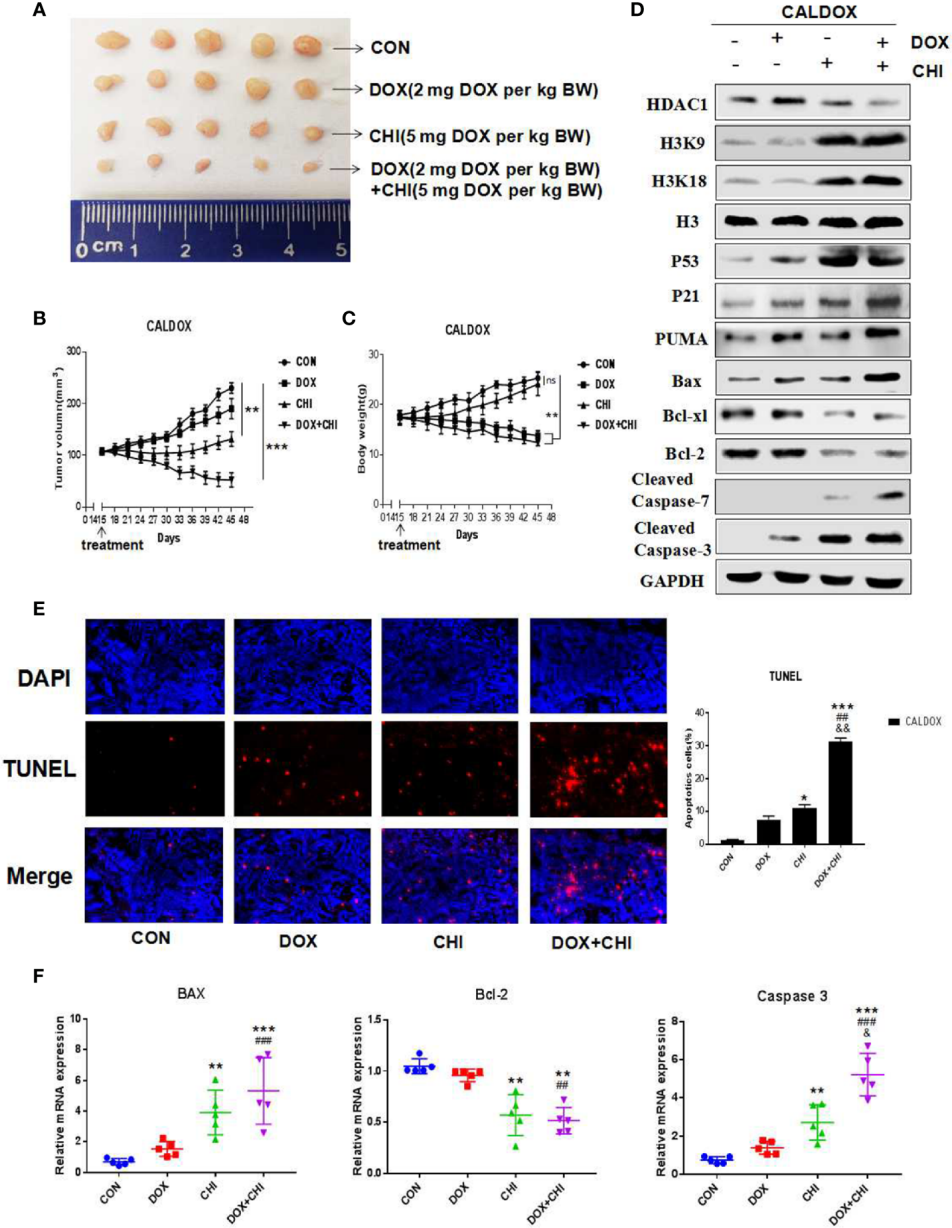
Antitumor activity of chidamide (CHI) and/or doxorubicin (DOX) in MDA breast cancer cells *in vivo.* **(A–C)** CALDOX xenograft tumor growth curve, size, and body weight after treatment with normal saline (control), DOX, CHI or CHI + DOX. **(D)** Western blot analysis of HDAC1, H3K9, H3K18, p53, p21, Puma, Bcl-xl, Bcl-2, cleaved caspase-7, cleaved caspase-3, and Bax on CALDOX-derived tumors treated with PBS (control), DOX, CHI, or CHI + DOX. **(E)** TUNEL staining analyzed cell apoptosis after treatment with normal saline (control), DOX, CHI, or CHI + DOX. **(F)** Relative fold change of Bax, Bcl-2, and caspase-3 gene expression levels in CALDOX-derived tumors treated with normal saline (control), DOX, CHI, or CHI + DOX. The numerical values are showed as mean ± SD of three independent replicates. “*” indicates a significant difference compared with the control group (*P < 0.05, **P < 0.01,***P<0.001),”#” indicates a significant difference compared with the DOX-treated group (^##^P < 0.01, ^###^P < 0.001), and “&” indicates a significant difference compared with the CHI-treated group (^&^P < 0.05, ^&&^P < 0.01). ns, no significance.

CHI combined with DOX had significant antitumor activity *in vitro* against MDR breast cancer cells, which prompted to study whether its antitumor effect *in vivo* could be maintained. The CALDOX cells were injected into the mammary fat pad of female nude mice. On the 14th day after injection, the mice were randomly divided into four groups, with an equal number of mice in each group. Each group was treated with DOX, CHI, CHI + DOX or vehicle control (**Figure 5A**). As expected, the tumors in the DOX-treated group continued to grow in the xenograft models, indicating DOX resistance. Tumor progression reduced to a certain extent in the CHI-treated group compared with the control and DOX-treated groups. However, the combined treatment group showed a more significant reduction in tumor growth in the MDR xenograft model (**Figure 5B**). Animals in the DOX-treated group and the combined treatment group lost significant body weight (BW). On the contrary, no significant loss of BW was observed in the CHI-treated group in the MDR xenograft model (**Figure 5C**). Consistent with the *in vitro* results, the western blot results showed that compared with the control group, the level of Bcl-xl, Bcl-2 were downregulated and those of p53, p21, Puma, cleaved caspase-7, cleaved caspase-3, and Bax were upregulated in the combined treatment group (**Figure 5D**). The percentage of TUNEL-positive cells was significantly higher in the combined treatment group than in the monotherapy and the control groups (**Figure 5E**). RT-qPCR results showed that compared with the control group, the relative gene expression of Bax and caspase-3 was significantly increased, while the relative gene expression of Bcl-2 was decreased (**Figure 5F**).

## Discussion

Chemotherapy is the preferred treatment for breast cancer. Cancer cells become resistant to drugs over time, which is a major cause of chemotherapy failure and disease recurrence (25–27). DOX is an important chemotherapy drug in the treatment of breast cancer. Drug resistance is a complex phenomenon involving multiple mechanisms (26, 28). New methods are urgently needed to avoid or slow down the occurrence of drug resistance so as to improve the efficacy of chemotherapy. Two drug-resistant breast cancer cell lines, MCF-7/A02 and CALDOX, were used in this study. Both cell lines had MDR phenotypes, but the mechanisms were different. Previous studies proved that the most important factor of MCF-7/A02 resistance was the overexpression of P-gp, and the resistance mechanism of CALDOX cells did not depend on the drug transporter. Although the reasons for drug resistance were different in these cell lines (20), HDAC1 was activated in the two MDR cell lines, which was consistent with the results reported by other organizations (29, 30). CHI is the international first subtype-selective HDACi independently developed by MicroCore Biology. It is mainly used for various types of lymphocyte or myelogenous leukemia (31). CHI has been used in various clinical and preclinical studies in recent years, In 2019, it was approved in combination with isetam for hormone receptor-positive advanced breast cancer (18). Therefore, the inhibition of HDAC is a new therapeutic approach. The clinical and basic research on the use of CHI in the treatment of breast cancer is ongoing (32–34). This study was done *in vivo* and *in vitro* experiments. The results revealed that the expression of HDAC1 was higher in resistant cells than sensitive cells. Therefore, two drug-resistant breast cancer cell lines CALDOX and MCF-7/A02 were used as the research objects to explore the effects of inhibition on the proliferation and apoptosis of drug-resistant breast cancer cells. CHI significantly increased the histone H3 acylation level of drug-resistant cells and reduced the expression of HDAC1 regardless of the addition of DOX. This was consistent with recent findings that CHI treatment increased the expression of Lys18 of H3 acetylation in myeloid leukemia K562 and ThP-1 cells, and the expression of Lys9 and Lys18 of H3 acetylation in human myeloma RPMI-8226 and ARP-1 cells (23, 35). Drug-resistant cloning experiments and EDU experiments showed that single-drug CHI had a certain effect on CALDOX and MCF-7/A02 cell proliferation. However, The combination significantly inhibited cell proliferation.

CHI induces cell cycle arrest in several ways, the most important of which seems to be the increase in cell cycle gene expression. The cell cycle distribution results showed that irrespective of the addition of DOX, CHI could cause G0/G1 cell cycle arrest and inhibit cell growth, which might be related to the up-regulation of the expression of p21. This was consistent with the results of previous research. HDAC1 promotes the expression of p21 to a certain extent. The p53 bound to the C-terminal Sp1 of p21, the region where p53 and HDAC1 competed for binding. After HDACi treatment, HDAC1 was released from the p21 promoter Sp1-binding site, inhibiting deletion and transcription induction, thereby increasing the expression of p21 and arresting the cells in the G0/G1 phase (36, 37). At the same time, recent studies found that CHI regulated TS genes through miR-129-3p, resulting in the G1-phase arrest of non-small cell lung cancer (NSCLC) H1355 and A549 cells. In myeloid plastic syndrome SKM-1, Mutz-1 cells and leukemia KG-1 cells, CHI blocks cell cycle in the G0/G1 phase by upregulating of the expression of p21 (24, 38).

The induction of apoptosis has been shown to be a promising way for the development of new anticancer drugs. Previous studies showed that HDACi (CHI, MS-275/FK228, panobinostat, quisinostat, sodium butyrate) induced caspase cascade by activating apoptotic intrinsic pathways and increasing mitochondrial permeability (39–43). In this study, flow cytometry and TUNEL staining results showed that CHI enhanced the number of cell apoptosis in drug-resistant cells. In drug-resistant cells, p53, as a tumor suppressor, remained silent, while p53 protein expression was upregulated in the CHI-treated and combined treatment group, which might be the cause of cell apoptosis. Activated p53 upregulated the recombinant protein of apoptotic regulator factor (Puma), downregulated the anti-apoptotic protein Bcl-xl and Bcl-2, and activated the pro-apoptotic protein Bax. When regulated by p53 signal, it was transferred from the cytoplasm to mitochondria, bound to the mitochondrial membrane and released cytochrome C. Under the action of dATP, cytochrome C was released into the cytoplasm, combined with apoptotic protease activator 1 (APAF-1) to form a polymer, and combined with the precursor of caspase 9 to form apoptosomes, and caspase 9 was activated. The caspase 9 activated a series of caspase members downstream of the pathway, including caspase 7 and caspase 3, further inducing specific apoptotic substrates and cell apoptosis.

## Conclusion

In summary, CHI combined with DOX can synergistically inhibit cell proliferation, reduce HDAC1 expression, activate p53, release p21, cause G0/G1 cell cycle arrest, and initiate apoptosis signaling pathway. This might be one of the important mechanisms for CHI combined with DOX to reverse drug resistance in breast cancer. This study provided evidence to support the efficacy and safety of CHI *in vitro* and *in vivo* in suppressing drug resistance in the treatment of breast cancer (44).

## Data Availability Statement 

The raw data supporting the conclusions of this article will be made available by the authors, without undue reservation.

## Ethics Statement

The animal study was reviewed and approved by Animal Care and Use Committee of Tianjin Cancer Hospital.

## Author Contributions

JZ and SZ conceived and designed the study. LC and QY performed the experiments. ZS and JL analyzed experimental results. LC, TP, and DZ wrote the draft of the manuscript. SZ reviewed and edited the manuscript. All authors contributed to the article and approved the submitted version.

## Funding

This study was supported by research funding from the National Natural Science Foundation of China (81502306, 81672623); Tianjin Municipal Science and Technology Committee (19YFZCSY00030); General project of scientific research program of Tianjin Municipal Education Commission (2019KJ186).

## Conflict of Interest

The authors declare that the research was conducted in the absence of any commercial or financial relationships that could be construed as a potential conflict of interest.

## Supplementary Material

Supplementary Figure 1
**(A)** Effect of CHI monotherapy on cell cycle. With the increase in concentration, the inhibition of the G0/G1 phase was also enhanced. **(B)** Effect of CHI on acetylation of H3. As the concentration increased, the acetylation of H3K9 and H3K18 also increased.

## Abbreviations

MDR, multidrug-resistant; HDAC, histone deacetylase; HDACi, histone deacetylase inhibitor; CHI, chidamide; DOX, doxorubicin.
