# Correction: Chidamide combined with doxorubicin induced p53-driven cell cycle arrest and cell apoptosis reverse multidrug resistance of breast cancer

**DOI:** 10.3389/fonc.2025.1722174

**Published:** 2026-01-12

**Authors:** Lixia Cao, Shaorong Zhao, Qianxi Yang, Zhendong Shi, Jingjing Liu, Teng Pan, Dongdong Zhou, Jin Zhang

**Affiliations:** Third Department of Breast Surgery, Tianjin Medical University Cancer Institute and Hospital, National Clinical Research Center for Cancer, Key Laboratory of Cancer Prevention and Therapy, Clinical Research Center for Cancer, Tianjin, China

**Keywords:** breast cancer, histone deacetylase, chidamide, doxorubicin, drug resistance

There was a mistake in “**Figure 1** and **Figure 2**” as published. In “**Figure 1E**”, the Western blot image for GAPDH was incorrectly uploaded. In “**Figure 2B, C**”, the mistake was caused by an unintentional assembly mistake. In “**Figure 2B**”, an inadvertent image duplication occurred between the CON and DOX groups in the CALDOX cell line. In “**Figure 2C**”, it has come to our attention that the data presented were inadvertently taken from a different experimental replicate. The corrected “**Figure 1** and **Figure 2**” appears below.

**Figure 1:**
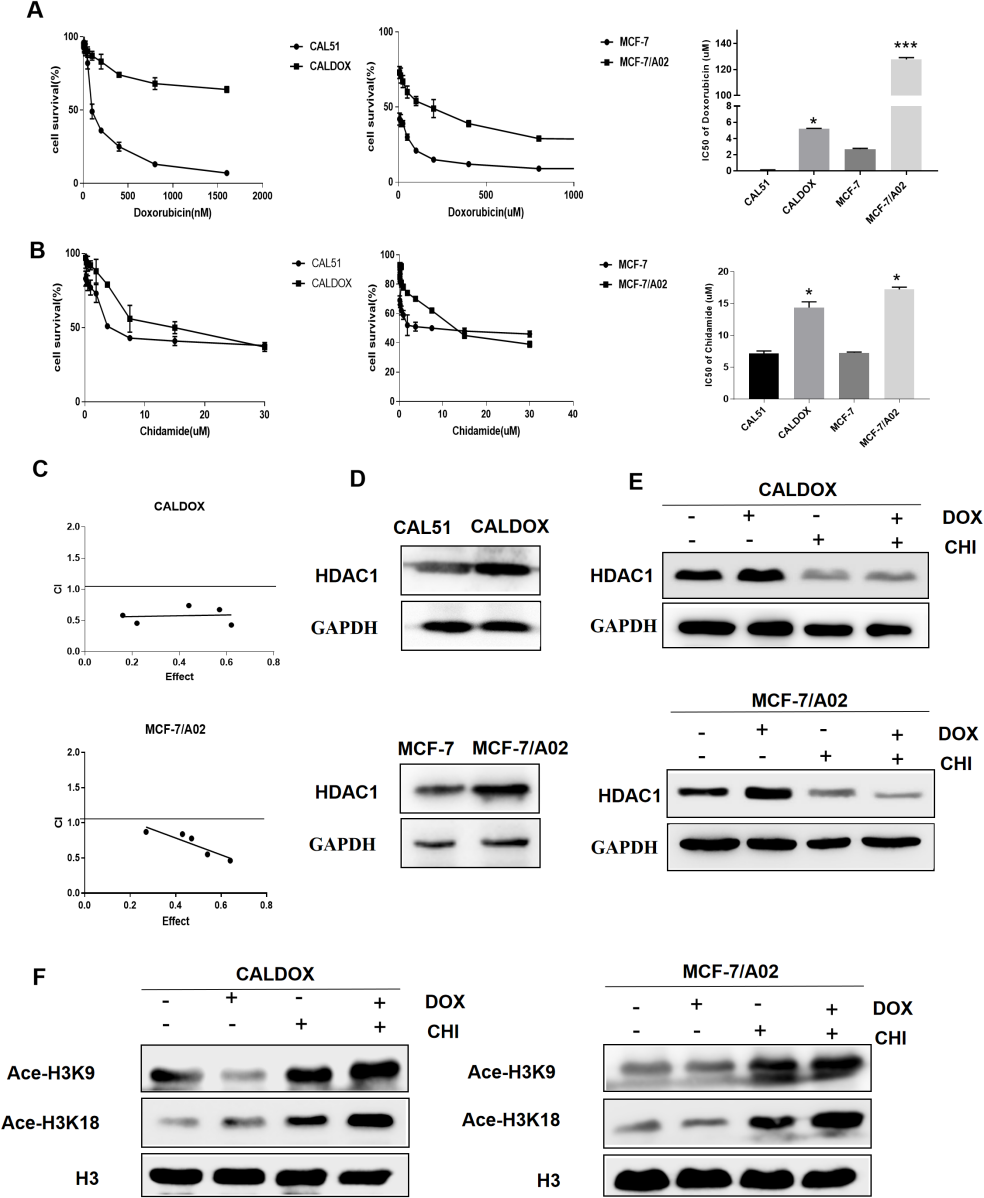
Effects of chidamide (CHI) and/or doxorubicin (DOX) on the viability and histone H3 acetylation of MDR breast cancer cells. **(A)** IC50 values of DOX of two pairs of human breast cancer cell lines and their multidrug-resistant (MDR) sublines. **(B)** IC50 values of CHI of two pairs of human breast cancer cell lines and their MDR sublines. **(C)** Cytotoxicity of CHI and DOX to CALDOX and MCF-7/A02 cells. **(D)** Expression of HDAC1 in sensitive and resistant cell lines. **(E)** Effects of CHI and DOX on HDAC1 expression in drug-resistant cells. **(F)** Effects of CHI and DOX on acetylation of H3K9 and H3K18 in drug-resistant cells. H3 was used as a loading control. The numerical values are expressed as mean ± standard deviation (SD) of three independent replicates. *P < 0.05, ***P < 0.001.

**Figure 2:**
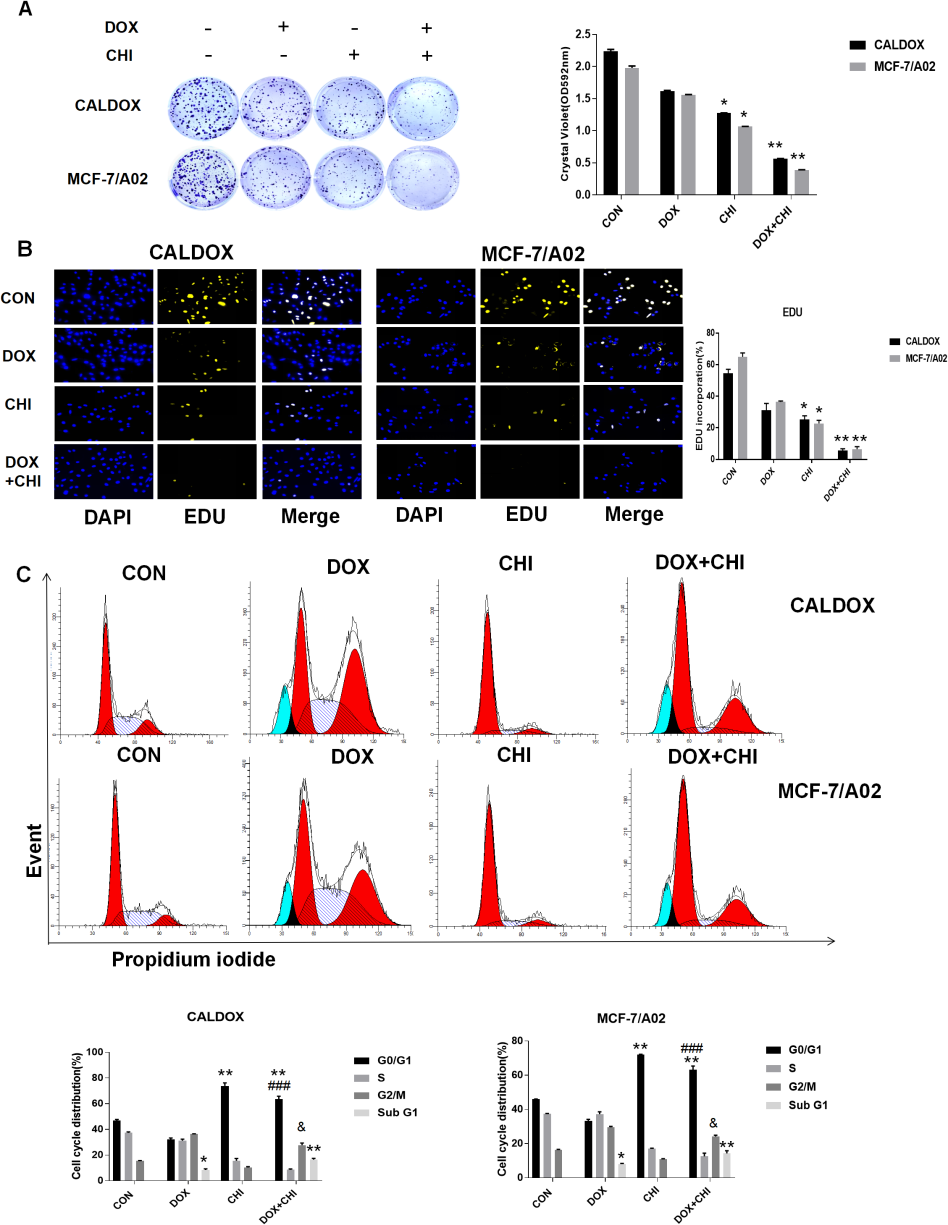
Effects of chidamide (CHI) and/or doxorubicin (DOX) on the proliferation and cell cycle of multidrug-resistant (MDR) breast cancer cells. **(A)** Drug resistance clonogenic assay confirmed the effect of CHI and/or DOX on cell proliferation. **(B)** EDU staining confirmed the effect of CHI and/or DOX on cell proliferation. **(C)** Effects of CHI and/or DOX on cell cycle. Numerical values are expressed as mean ± SD of three independent replicates. “*” indicates a significant difference compared with the control group (*P < 0.05, **P < 0.01), ”^#^” indicates a significant difference compared with the DOX-treated group (^###^P<0.001), and “&” indicates a significant difference compared with the CHI-treated group (^&^P<0.05).

The original version of this article has been updated.

