# Supplementary figures and images for: Chidamide Combined With Doxorubicin Induced p53-Driven Cell Cycle Arrest and Cell Apoptosis Reverse Multidrug Resistance of Breast Cancer
Source: Front Oncol. 2021 Mar 2;11:614458. doi: 10.3389/fonc.2021.614458 (PMC7962870; doi:10.3389/fonc.2021.614458)

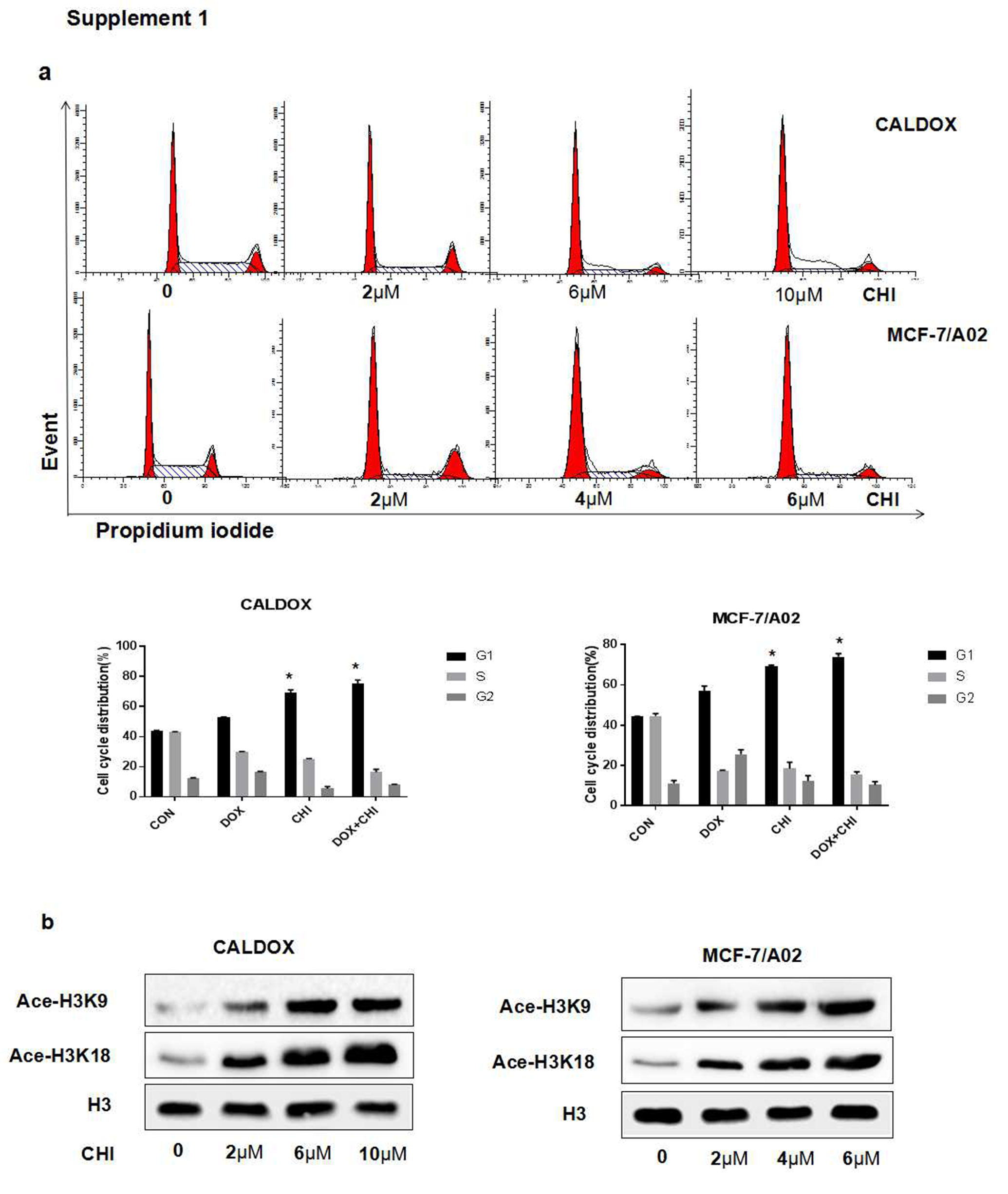

Supplement: Supplementary Figure 1 — (A) Effect of CHI monotherapy on cell cycle. With the increase in concentration, the inhibition of the G0/G1 phase was also enhanced. (B) Effect of CHI on acetylation of H3. As the concentration increased, the acetylation of H3K9 and H3K18 also increased. [file Image_1.tif]
